# Chemosymbiotic bivalves contribute to the nitrogen budget of seagrass ecosystems

**DOI:** 10.1038/s41396-019-0486-9

**Published:** 2019-08-08

**Authors:** Ulisse Cardini, Marco Bartoli, Sebastian Lücker, Maria Mooshammer, Julia Polzin, Raymond W. Lee, Vesna Micić, Thilo Hofmann, Miriam Weber, Jillian M. Petersen

**Affiliations:** 1 University of Vienna, Centre for Microbiology and Environmental Systems Science, Division of Microbial Ecology, Vienna, Austria; 2 Integrative Marine Ecology Department, Stazione Zoologica Anton Dohrn, National Institute of Marine Biology, Ecology and Biotechnology, Napoli, Italy; 3 Marine Research Institute, University of Klaipeda, Klaipeda, Lithuania; 4 Department of Life Sciences, University of Parma, Parma, Italy; 5 Department of Microbiology, Institute for Water and Wetland Research, Radboud University, Nijmegen, The Netherlands; 6 School of Biological Sciences, Washington State University, Pullman, WA USA; 7 University of Vienna, Centre for Microbiology and Environmental Systems Science, Department of Environmental Geosciences, Vienna, Austria; 8 HYDRA Marine Sciences GmbH, Sinzheim, Germany; 9 HYDRA Field Station Elba, Campo nell’Elba (LI), Italy; 10 Present Address: Department of Environmental Science, Policy, and Management, University of California, Berkeley, CA USA

**Keywords:** Biogeochemistry, Stable isotope analysis, Microbial ecology, Stable isotope analysis, Environmental microbiology

## Abstract

In many seagrass sediments, lucinid bivalves and their sulfur-oxidizing symbionts are thought to underpin key ecosystem functions, but little is known about their role in nutrient cycles, particularly nitrogen. We used natural stable isotopes, elemental analyses, and stable isotope probing to study the ecological stoichiometry of a lucinid symbiosis in spring and fall. Chemoautotrophy appeared to dominate in fall, when chemoautotrophic carbon fixation rates were up to one order of magnitude higher as compared with the spring, suggesting a flexible nutritional mutualism. In fall, an isotope pool dilution experiment revealed carbon limitation of the symbiosis and ammonium excretion rates up to tenfold higher compared with fluxes reported for nonsymbiotic marine bivalves. These results provide evidence that lucinid bivalves can contribute substantial amounts of ammonium to the ecosystem. Given the preference of seagrasses for this nitrogen source, lucinid bivalves’ contribution may boost productivity of these important blue carbon ecosystems.

## Introduction

Shallow-water chemosynthetic symbioses are widespread where decomposition of organic matter produces sulfide [1]. However, their relevance for ecosystem functioning has received limited attention due to the assumption that chemosynthesis plays a minor role in shallow-water ecosystems. Recent studies are challenging this assumption [2–4]. In seagrass sediments, bivalves of the family Lucinidae consume sulfide through their chemosynthetic symbionts, allowing more plant growth while relying on the seagrass to stimulate sulfide production by free-living sulfate-reducing microorganisms [3]. Still, we know little about nutrient cycling in lucinid bivalves at both the organism and the ecosystem scale. Most studies to date have focused on carbon (C) fixation by the symbionts and transfer to the host [5, 6] or on the additional contribution of filter feeding to host nutrition [7]. Nitrogen (N) metabolism has received far less attention until recently, when dinitrogen (N_2_) fixation by chemosynthetic symbionts was shown to be possible in two lucinid species [8, 9]. Concurrently, chemosynthetic symbioses can, to varying degrees, gain their N from ammonium (NH_4_^+^), nitrate, or dissolved free amino acids in their environment [10–12], with the symbionts being able to recycle N waste compounds within the symbiosis [13]. Surprisingly, although these studies attest to the expanded N metabolic versatility of chemosynthetic symbioses, the significance of lucinid bivalves in contributing to their ecosystem N budget has been largely overlooked. Since the lucinid symbionts have a versatile C and N metabolic repertoire, being able to fix inorganic C or grow heterotrophically, and to take up various nitrogen forms [8], they might provide lucinid bivalves with a distinct advantage over nonsymbiotic filter-feeding bivalves, while boosting their role in the biogeochemistry of seagrass ecosystems.

## Methods

We studied a lucinid bivalve (*Loripes orbiculatus*) in the seagrass (*Posidonia oceanica*) sediments of Elba Island (Italy) during two field expeditions in April (spring) and October (fall) 2016. *P. oceanica* tends to consume porewater nutrients (particularly nitrogen) during the growth phase (spring and summer), which are therefore depleted in fall, while sulfide accumulates as a result of leaf burial and decomposition. To check if this was true for our study site, we analyzed porewater inorganic nutrient concentrations (dissolved inorganic nitrogen—DIN and dissolved inorganic phosphorus—DIP) down to 60 cm below the sediment surface, with a resolution of 5 cm. Stable isotope probing with ^13^C–NaHCO_3_^−^ and ^15^N–N_2_ was used to quantify C and N_2_ fixation by the chemosynthetic symbionts. Exogenous sulfide was not added to the incubation seawater as our primary goal was to investigate environmentally driven differences in physiology in both seasons. An isotope pool dilution (IPD) experiment with ^15^N–NH_4_Cl, was conducted in October to quantify gross and net NH_4_^+^ fluxes by the bivalve symbiosis. The IPD technique has not yet been applied in marine symbiosis research. This technique involves labeling the nutrient pool of interest (in our case by adding ^15^NH_4_^+^). By quantifying the relative proportion of heavy and light isotopes in the nutrient pool, and the change in concentration over time, gross production (i.e., mineralization) and consumption (i.e., immobilization) rates can be calculated. Finally, elemental and natural stable isotope analyses (*δ*^13^C and *δ*^15^N, C:N ratio; symbiotic tissue mass index, SMI; and gill total S content) were carried out to study the stoichiometric and isotopic niche (as proxies of the ecological niche) of host and symbiont under the two contrasting seasons. Individual *δ*^13^C and *δ*^15^N values of symbiont-bearing and nonsymbiotic tissues were analyzed to compare isotopic niche spaces of symbionts and host in April and October. For more details on all methods see the Supplementary Methods.

## Results and discussion

The biogeochemistry of *P. oceanica* sediments is highly influenced by the seagrass seasonal growth, leaf burial, and decay by microorganisms. *P. oceanica* growth shows a late spring maximum and a fall minimum [14]. The plant tends to consume porewater nutrients (particularly nitrogen) during the growth phase (spring and summer), which are therefore depleted in fall, while sulfide accumulates as a result of leaf burial and decomposition [15]. Our porewater profiles confirm this pattern, with higher DIN concentrations and DIN:DIP ratios in April compared with October (*p* < 0.01; Fig. S1).

**Fig. 1:**
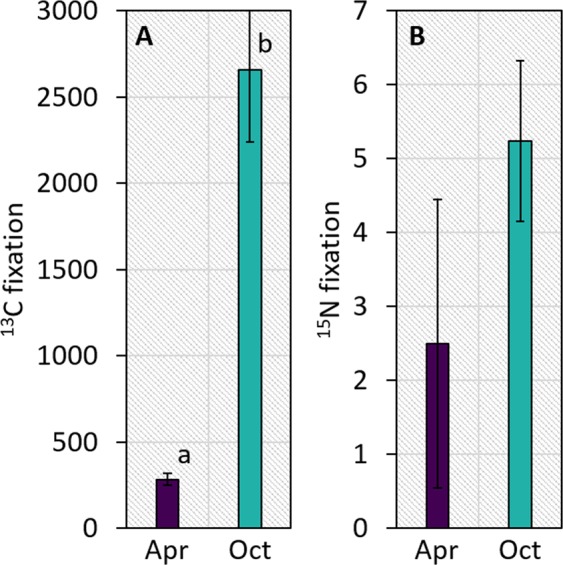
Results from ^13^C–HCO_3_^−^ and ^15^N–N_2_ isotope probing experiments: **a** Carbon and **b** dinitrogen fixation by the microbial symbionts (nmol **C** (or N) g gill tissue^−1^ h^−1^ ± SE, n = 5). Sampling points are color-coded in purple (April) and cyan (October). Different lowercase letters indicate significant differences (*p* < 0.05, PERMANOVA)

*L. orbiculatus* is able to supplement its diet with filter feeding on a seasonal basis [7]. Here we show that not only the host, but also the chemoautotrophic symbionts may modulate their metabolic activities according to the availability of external (or recycled) resources. C fixation by the symbionts was roughly 10-fold higher in October compared with April (*p* < 0.001; Fig. 1a). N_2_ fixation, measured for the first time here in a chemosynthetic symbiosis using the ^15^N–N_2_ method, also increased in October, although not significantly (Fig. 1b). The boost in autotrophy was potentially mediated by higher sulfur energy storage within the symbionts (*p* < 0.01; Fig. S4).

**Fig. 2:**
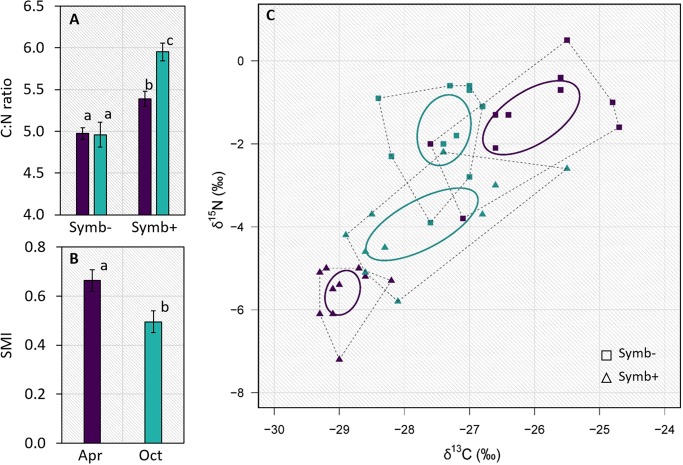
Results from freshly sampled bivalve specimens: **a** C:N ratio (± SE, *n* = 10) of symbiont-free (Symb-) and symbiont-hosting (Symb+) animal tissues; **b** Symbiotic tissue mass index—SMI. The SMI indicates the proportion of symbiont-hosting gill biomass (mg mm^−1^ ± SE, *n* = 10; see Supplementary Methods for details on how this index was calculated); **c** Biplot of the natural abundance of ^13^C and ^15^N isotopes showing the total amount of niche space occupied (total area, dashed polygons) and the isotopic niche width (standard ellipse area, solid ellipses) as proxies of trophic specialization of symbiont-free (squares) and symbiont-hosting (triangles) animal tissues. Sampling points are color-coded in purple (April) and cyan (October). Different lowercase letters indicate significant differences (*p* < 0.05, PERMANOVA)

The increased C fixation rates drove the C:N ratio of the symbionts higher, but not of the host (*p* < 0.001; Fig. 2a), attesting to the stoichiometric flexibility of the autotrophic partner in the symbiosis and the homeostasis of the heterotrophic host [16]. However, the distribution of bi-variate Bayesian ellipses shows that the natural isotopic niche of the sulfide-oxidizing symbionts was significantly larger in the samples collected in October (Fig. S2; i.e., lower trophic specialization), which may indicate a history of mixotrophic metabolism of the endosymbionts, consistent with the presence of a complete tricarboxylic acid cycle and transporters for uptake of organic compounds in their genome [8].

The proportion of symbiont-hosting gill biomass (SMI) was lower in October (*p* < 0.05; Fig. 2b). At the same time, there was a strong overlap in C isotopic niche of host and symbionts, indicating a match in their C source, while there was a mismatch in April (Fig. 2c). These results could be explained by a flexible nutritional mutualism. Under nutrient rich/high productivity conditions in April, when labile organic matter in seagrass sediments is highest [17], the host relies more on mixotrophy through filter feeding. Under nutrient depleted/low productivity but sulfide-rich conditions in October, the symbiosis shifts toward relying more on the symbionts as a source of energy. Our observation that symbiont C fixation rates were ten times higher in October compared with April is consistent with this theory.

Filter-feeding bivalves that do not host chemosynthetic symbionts enter a “dormant” state in summer, possibly due to food limitation [18 and references therein]. The ability to harvest energy throughout the summer and fall by relying on symbiont primary production when food availability is low would provide lucinid bivalves with a distinct advantage over nonsymbiotic filter-feeding bivalves. While more targeted approaches will be needed to conclusively verify this hypothesis, gross NH_4_^+^ production and consumption measured in October using IPD indicated that the symbiosis was indeed C limited, as bivalves consumed NH_4_^+^ only when exposed to a source of labile organic C (Fig. S3).

The same experiments, using IPD on an invertebrate symbiotic animal for the first time to our knowledge, allowed us to quantify gross and net excretion rates contributed by the symbiosis to its surroundings. Net excretion by the bivalves was ~15 µmol NH_4_^+^ g_SFDW_^−1^ h^−1^ (Fig. S3), which is up to tenfold higher compared with NH_4_^+^ excretion rates reported for other nonsymbiotic marine bivalves [19] and testifies to the potential of these chemosynthetic symbioses to underpin ecosystem functioning by nitrogen provisioning.

## Conclusions

In this study, we show that *L. orbiculatus* likely has a flexible nutritional mutualism, in which host and symbionts cycle between a looser trophic association and a tight chemoautotrophic partnership, changing nutritional strategy according to the environmental conditions. Further, we report that under C-limiting conditions these chemosymbiotic bivalves can excrete substantial amounts of NH_4_^+^ to the environment. In seagrass sediments, lucinids and their endosymbionts are not only relevant for their role in sulfide detoxification [3], but can also provide the plant’s preferred N form [20], thus contributing to the productivity of these important blue carbon ecosystems.

## Supplementary information

Supplementary figures

Supplementary text
