# Supplementary material for: Chemosymbiotic bivalves contribute to the nitrogen budget of seagrass ecosystems
Source: ISME J. 2019 Aug 8;13(12):3131–4. doi: 10.1038/s41396-019-0486-9 (PMC6863832; doi:10.1038/s41396-019-0486-9)
Supplement: Supplementary file 1 — Supplementary figures [file 41396_2019_486_MOESM1_ESM.pdf]

Chemosymbiotic bivalves contribute to the nitrogen budget of seagrass ecosystems

Cardini U, Bartoli M, Lücker S, Mooshammer M, Polzin J, Lee R, Micić V, Hofmann T, Weber M, Petersen JM

Supplementary figures

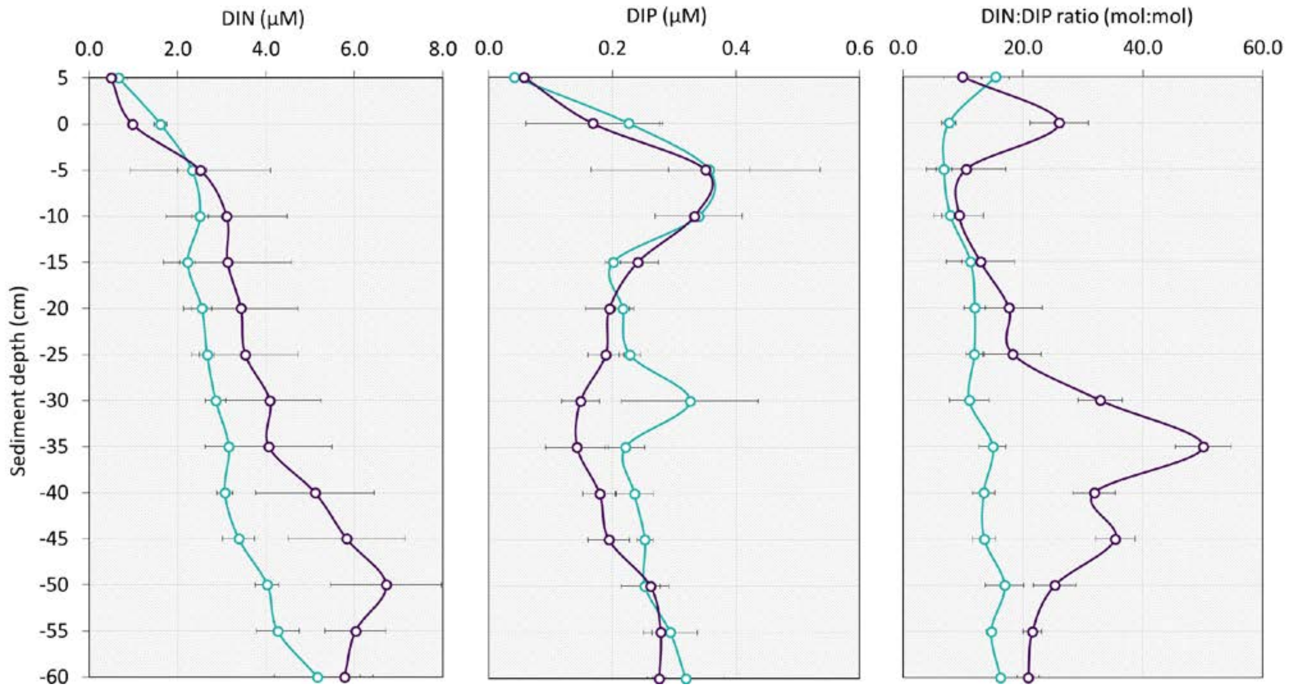

Figure S1: Dissolved inorganic nutrients and DIN:DIP ratio in the porewater at the time and place of sampling (± SE, n = 3), color-coded in purple (April) and cyan (October).

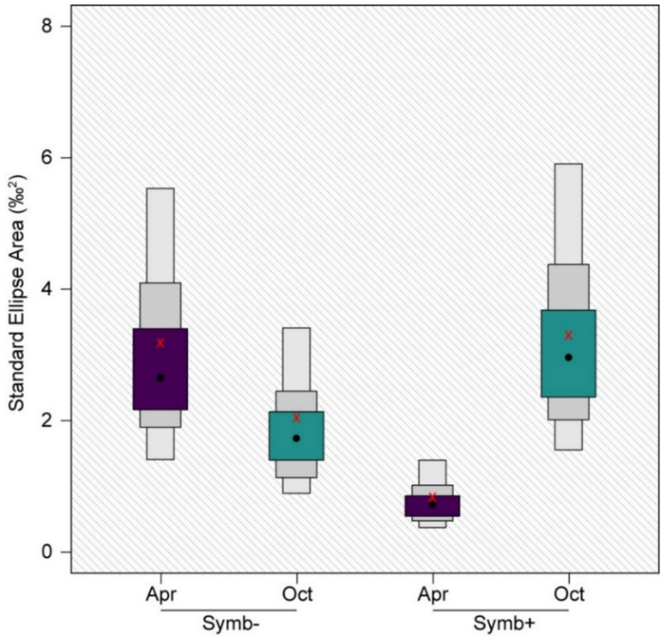

Figure S2: Distribution of Bayesian ellipses showing the isotopic niche width (as a proxy of trophic specialization) and its uncertainty for symbiont-free (Symb-) and symbiont-hosting (Symb+) animal tissues (freshly sampled bivalve specimens), color-coded in purple (April) and cyan (October). Black dots represent the mode while red crosses are group means and the shaded boxes represent the 50%, 75% and 95% credible intervals from dark to light grey.

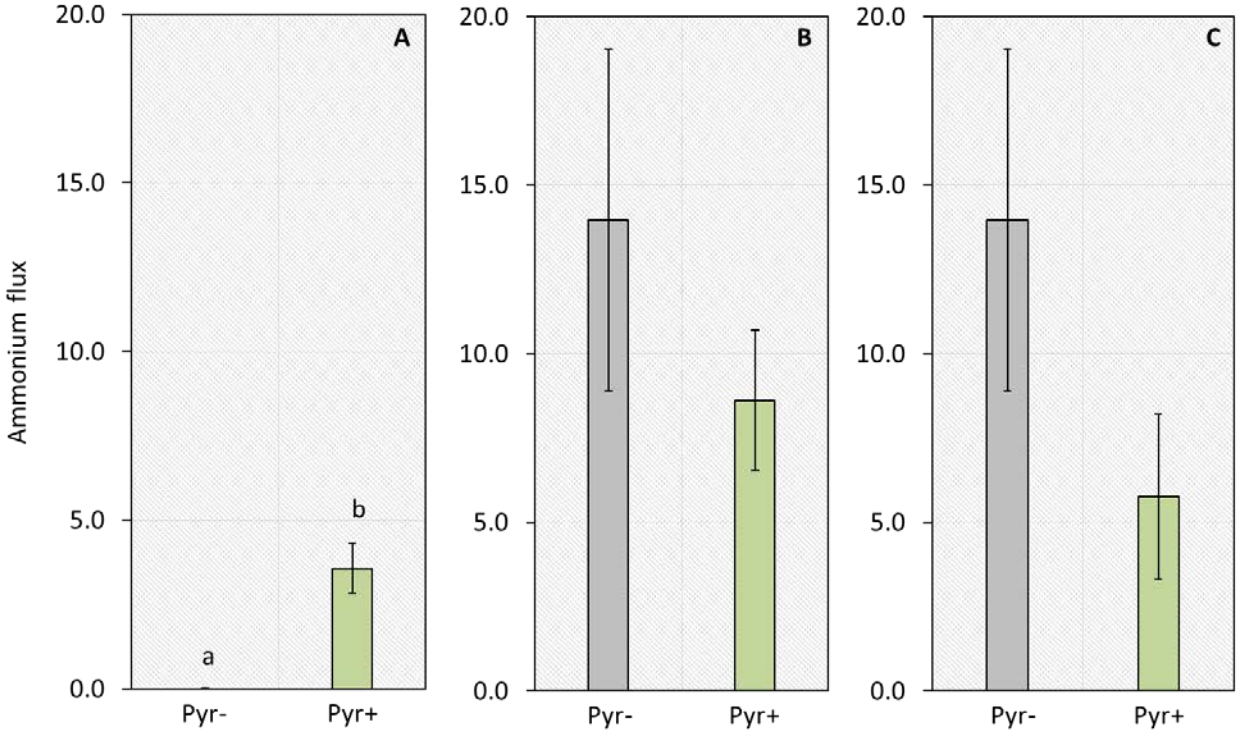

Figure S3: Ammonium fluxes (µmol NH_4^+ g^{-1} h^{-1} ± SE, n = 5) driven by the bivalve symbiosis, measured during the isotope pool dilution experiment in October. A) Gross uptake; B) Gross excretion; C) Net excretion. Results for the incubations with natural filtered seawater (Pyr-) are reported in grey, while results of the incubations amended with 10 µM pyruvate (Pyr+) are reported in green. Different lowercase letters indicate significant differences (p < 0.01, PERMANOVA).

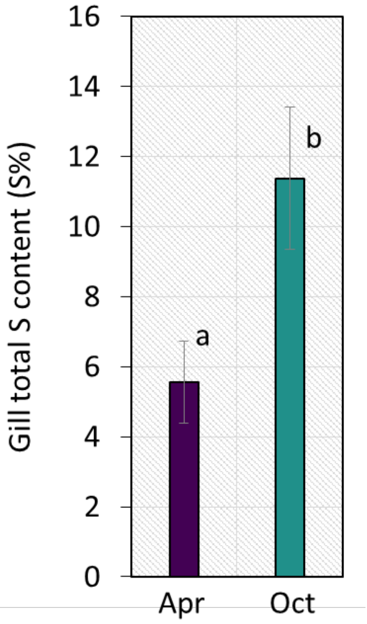

Figure S4: Gill total S content (S%) of freshly sampled bivalve specimens collected during the two field expeditions. Results are color-coded in purple (April) and cyan (October). Different lowercase letters indicate significant differences (p < 0.01, PERMANOVA).
