# Supplementary material for: Chemosymbiotic bivalves contribute to the nitrogen budget of seagrass ecosystems
Source: ISME J. 2019 Aug 8;13(12):3131–4. doi: 10.1038/s41396-019-0486-9 (PMC6863832; doi:10.1038/s41396-019-0486-9)
Supplement: Supplementary file 2 — Supplementary text [file 41396_2019_486_MOESM2_ESM.pdf]

Chemosymbiotic bivalves contribute to the nitrogen budget of seagrass ecosystems

Cardini U, Bartoli M, Lücker S, Mooshammer M, Polzin J, Lee R, Micić V, Hofmann T, Weber M, Petersen JM

Supplementary text

Field collection and porewater nutrients

All the sampling activities and the incubation experiments were conducted during two field expeditions to the Elba Island station of the HYDRA Institute for Marine Sciences in Fetovaia, Livorno (Italy) in April and October 2016. Specimens of *Loripes orbiculatus* were collected by scuba diving in the bay of Fetovaia from sediments adjacent to a *Posidonia oceanica* meadow (42°43'48"N 10°9'23"E) at approximately 7 m depth (seawater temperature: 16 ± 0.5 °C in April, 20 ± 0.5 °C in October). The bivalves were transported back to the Station with a good quantity of their surrounding sediment within 30 minutes and placed in the shade at ambient temperature in 30 L aquaria with natural seawater to acclimatize before the incubation experiments.

The NW Mediterranean Sea typically shows the sequence of events characteristic of a temperate sea regime (i.e., fall-winter mixing, spring primary production, and stratification in summer) [1]. Inorganic nutrient concentrations generally increase during winter/spring, when seawater temperature decreases causing deep mixing of the water column and the following spring phytoplankton bloom. The sedimentation of the phytoplankton bloom (low C/N ratio) makes these nutrients available to the benthos (e.g., [2]). Additionally, seagrass activity strongly influences dissolved nutrients availability in the porewater of seagrass sediments (e.g., [3]). In particular, the plant tends to consume porewater nutrients (particularly nitrogen) during the growth phase (spring and summer), which are therefore depleted in fall, while sulfide accumulates as a result of leaf burial and decomposition [4]. To check if this was true for our study site, porewater was collected by scuba divers at the sampling location in the bay using metered stainless steel lances designed to penetrate down to 60 cm below the sediment surface, with a resolution of 5 cm between collected samples. In each season, three profiles were collected for a total of 42 samples (14 x 3). Porewater samples were filtered on 0.22 µm polycarbonate membrane filters (Merck Millipore), preserved frozen at -20 °C and finally analyzed for nitrate, nitrite, ammonium, and orthophosphate concentrations on a Continuous Flow Autoanalyzer (Flowsys, Systea s.p.a.) at the Stazione Zoologica Anton Dohrn, Napoli, Italy (**Fig. S1**).

^{13}C-HCO_3^- and ^{15}N-N_2 isotope probing experiments and stable isotope analysis

Isotope probing experiments were conducted with lucinid bivalves to quantify C and N_2 fixation by the chemosynthetic symbionts during the two field campaigns (**Fig. 1**). All material used for the incubation experiments was soaked in 2 M HCl overnight and rinsed with MilliQ water before use. ^{15}N- and ^{13}C-enriched seawater was prepared before the incubation. ^{13}C-$NaHCO_3^-$ (98 atom% 13C, Sigma Aldrich) was dissolved in 0.2-µm-filtered seawater to reach a $H^{13}CO_3^-$ concentration of 2 mM (final ^{13}C-atom% = 51.6%). ^{13}C-enriched seawater (or unamended natural abundance seawater, for the controls) was transferred to 250 ml serum bottles, crimp-sealed gas-tight with a hollow needle in the septum to prevent air bubbles forming. Thereafter, 10 ml of ^{15}N-N_2 gas (99 atom% ^{15}N, Cambridge Isotope Laboratories, lot number: 01/071401) (or of air, for the controls) were injected in the serum bottles with a hollow needle in the septum to allow replacement of the liquid. The procedure was repeated again with additional 20 ml of gas without the second needle in the septum, to create over-pressure within the bottles and aid dissolution of the gas [5, 6]. This modified procedure, without further dilution, resulted in a final ^{15}N-atom% of 47.4% (for details of the measurement see the following). The serum bottles were shaken for 2 minutes and stored in the dark, upside down, until use. The ^{15}N-N_2 gas was checked for the presence of ^{15}N-nitrate or ^{15}N-ammonium at the Max Planck Institute for Marine Microbiology, Bremen, Germany, and was found to be free of contamination [7].

25 ml serum bottles were prepared with a layer of 1.5 cm of acid-washed glass beads (425-600 µm, Sigma Aldrich) at the bottom to simulate the sediment in which the bivalve burrows. Each serum bottle was then filled with the respective treatment seawater (control or enriched), gently transferring the enriched seawater to minimize loss of ^{15}N-N_2 gas. One bivalve of ca. 1 cm shell height (adult size) was added to each bottle, which was subsequently crimp-sealed gas tight avoiding the formation of bubbles. Five bivalves (i.e., five bottles) were incubated either in ^{15}N- and ^{13}C-enriched seawater or in control seawater. Additionally, four “dead controls” were incubated to control for isotopic contamination. We killed these clams by opening them and adding 0.5 mL of 4% PFA solution ca. 12 hours before the incubation started, and kept the samples in the fridge until adding them to chambers amended with ^{13}C-$NaHCO_3^-$ and ^{15}N-N_2 enriched seawater as described above. Exogenous sulfide was not added to the incubation seawater as we wanted to investigate environmentally-driven differences in physiology in both seasons. Initial samples (n = 3) were collected from each 250 ml serum bottle in 12 ml exetainers (Labco Limited), preserved with 50 µl of 0.25 M $HgCl_2$ solution and stored upside down in the dark until analysis. The isotopic composition of the N_2 gas in the enriched seawater was analyzed after headspace equilibration using gas chromatography (Agilent 6890 equipped with a Porapak Q column at 80 °C and

a TCD detector at 300 °C; Agilent Technologies, Santa Clara, CA, USA) combined with mass spectrometry (Agilent 5975c, quadruple inert MS) as in [8]. After 24 h, the incubation bottles were opened, the bivalves collected, measured for their shell length and dissected. Symbiont-bearing (gill) tissue and non-symbiotic (host) tissue (i.e., the remaining tissue after removal of the gills) were separated and stored at -20 °C. We measured oxygen concentrations using an optical oxygen meter (FireStingO_2, PyroScience GmbH, Germany) and verified that oxic conditions were maintained at ca. 40% saturation at the end of a 24 h incubation. Ten freshly sampled bivalve specimens were dissected and preserved without any incubation both in April and in October to determine the natural $^{13}C/^{12}C$ and $^{15}N/^{14}N$ ratios of the gill and host tissues. Frozen tissues were freeze-dried for 48 h before being ground to fine powder and weighed into tin capsules that were crimped manually. Samples were analyzed for carbon and nitrogen elemental composition (%) and isotope ratios ($\delta^{13}C$ and $\delta^{15}N$) by continuous flow isotope ratio mass spectrometry (IRMS) using a Costech elemental analyzer interfaced with a GV Instruments Isoprime IRMS. Measures of $\delta^{13}C$ and $\delta^{15}N$ of the samples were compared against a protein powder calibrated using NIST standards with a precision and accuracy of 0.3 and 0.5 ‰, respectively. ^{13}C (**Fig. 1A**) and $^{15}N_2$ (**Fig. 1B**) incorporation rates were calculated following [6, 9], with the equation:

$$\frac{\text{gill } ^{15}\text{N (or } ^{13}\text{C) atom\% excess}}{\text{medium } ^{15}\text{N (or } ^{13}\text{C) atom\% excess}} \times \frac{\mu\text{g gill N (or C)}}{\text{g gill ind}^{-1}} \times \frac{1}{\Delta t}$$

and expressed as nmol N (or C) g gill tissue^{-1} h^{-1} ± SE. When calculating our C and N_2 fixation rates, we considered as “labeled” (and thus used the values for the calculation) those samples that showed an atom% excess that was higher than 2 times the standard deviation of the atom% of the unlabeled control samples. The dead controls always showed atom% excess values not different from the standard deviation of the atom% of the unlabeled control samples, both for ^{13}C and for ^{15}N.

^{15}N-NH_4Cl isotope pool dilution experiment

In October, we conducted an isotope pool dilution experiment to quantify gross and net NH_4^+ fluxes by the bivalve symbiosis (**Fig. S3**). The ^{15}N isotope pool dilution (IPD) technique is a method originally established and later extensively used in soil biogeochemistry (see Di, et al. [10] for a review), but has not yet been applied in marine symbiosis research. This is surprising as it is the only available method that allows simultaneous measurement of gross production and consumption rates of key nutrients. This technique is based on labeling the ammonium pool by adding ^{15}N-labelled ammonium. The quantification of the decrease in the isotopic label and the change in concentrations over time allows to calculate the gross production (i.e., mineralization) and immobilization rates. ^{15}N-NH_4Cl (98 atom% ^{15}N,

Sigma Aldrich) was added to 0.2-µm-filtered seawater to reach a $^{15}N\text{-}NH_4^+$ concentration of 0.4 µM. Thereafter, the incubation experiment consisted of 3 different sets of 25 ml serum bottles (n = 5 per treatment/control) prepared with a layer of 1.5 cm of acid-washed glass beads as described above. The control consisted of serum bottles with $^{15}N\text{-}NH_4^+$-enriched seawater but without bivalves. In the second set, the bivalves were added. The third set was additionally amended with sodium pyruvate ($C_3H_3NaO_3$, Sigma Aldrich, final concentration: 10 µM). *L. orbiculatus* symbionts encode a complete tricarboxylic acid (TCA) cycle and transporters for uptake of organic compounds and thus have the potential for heterotrophic growth [11]. We therefore decided to add pyruvate to provide the bivalves with a source of labile organic C. Initial samples were taken from the stock solution used to fill each set of bottles, and after 6 h of incubation from each serum bottle. The samples were collected in 12 ml exetainers, preserved with 50 µl of 0.25 M $HgCl_2$ solution and stored upside down in the dark until analysis. Each sample was analyzed for total NH_4^+ concentrations (with a standard spectrophotometric technique) and for ^{15}N:^{14}N ratios of NH_4^+. To this purpose, samples in exetainers were degassed with helium, treated with a hypobromite-iodine solution to oxidize NH_4^+ to N_2, and analyzed on a membrane-inlet mass spectrometer (Bay Instrument). Gross immobilization (i, uptake) and mineralization (m, excretion) rates by the bivalve symbiosis were calculated using the equations by Kirkham & Bartholomew [12], with net excretion = m - i. For the control samples without bivalves, the immobilization and mineralization rates were not different from zero, because no dilution of the isotopic label over time occurred, due to lack of activity.

Elemental and natural stable isotope analyses

C% and N% data obtained after analysis of freshly sampled bivalve specimens at the EA-IRMS was used to calculate C:N ratios of the gill and host tissues (**Fig. 2A**). Gill total S content (S%) was quantified on subsamples from the same freshly sampled bivalve specimens using an Elemental Vario MACRO CHNS analyzer (Hanau, Germany), with a within laboratory determined relative standard deviation of ± 0.1%. (**Fig. S4**). Prior to the analysis, the instrument calibration was adjusted to the daily ambient conditions by means of daily factor determination with the calibration standard sulfanilamide. The Symbiotic tissue Mass Index (**Fig. 2B**) was determined as the ratio between the gill tissue dry weight of each bivalve specimen (mg) and its shell length (mm). Although not providing direct evidence, higher index values (i.e. larger symbiont-bearing organs) are suggestive of higher *in-hospite* symbiont densities. Individual $\delta^{13}C$ and $\delta^{15}N$ values of symbiont-bearing and non-symbiotic tissues were analyzed using a Stable Isotope Bayesian Ellipses in R (SIBER) model [13, 14] to compare isotopic niche spaces of symbionts and host in April and October.

Ecologists are increasingly using stable isotope analyses to investigate variation in resource and habitat use from the individual to the community level [15]. In particular, the isotopic niche has become an established concept in trophic ecology, because stable isotope ratios in consumer tissues are tightly linked to those in their diet [13]. Overall, the isotopic niche (i.e., the isotopic composition) is a potentially powerful way to investigate ecological niches [15, 16]. When primarily driven by consumer-resource interactions, the isotopic niche aligns closely with the trophic niche as defined by Bearhop, et al. [17]. Multivariate ellipse-based metrics calculated using Bayesian inference were developed by Jackson, et al. [13] to robustly investigate the isotopic niche width, a proxy for the extent of trophic diversity. The SIBER Bayesian model generates a bi-plot with standard ellipses, which contain approximately 40% of the data (**Fig. 2C**) while being insensitive to sample size and robust measures of isotopic niche width in comparative studies between groups [13]. The distribution of Bayesian Standard Ellipse Areas (SEA_B) can be subsequently plotted for each group as a density plot. The model also calculates the uncertainty (i.e., 50%, 75% and 95% credible intervals) of this distribution, the mean, and the mode (i.e., the value that appears most often) (**Fig. S2**).

These methods are not often used in microbial ecology, where little biomass is typically available and stable isotope labeling approaches are more common because they allow tracking of single population (or single cell) activities. However, the concept of the isotopic niche is potentially a powerful tool to investigate trophic interactions in marine mutualistic symbioses. For example, the study of species-specific host and symbiont standard ellipse areas was recently used to investigate the trophic ecology of the coral-dinoflagellate symbiosis [18]. To our knowledge, this method was not previously applied to chemosynthetic symbioses.

Statistical analyses

Differences in each parameter were assessed using univariate distance-based permutational nonparametric analyses of variance (PERMANOVA) [19]. Environmental variables (DIN, DIP, DIN:DIP) were normalized and analyzed for differences between seasons based on Euclidean distances using type I (sequential) sum of squares with 9999 unrestricted permutations of raw data. All other variables were square root transformed, and analyses were based on Bray Curtis similarities using type III (partial) sum of squares with 9999 unrestricted permutations of raw data. Uptake and excretion rates were tested for differences between treatments (with or without pyruvate amendment), while the Symbiotic tissue Mass Index, the gill S content, C fixation and N fixation rates were tested for differences between seasons. A fully crossed design with two fixed factors (season, tissue) was used to test for

differences in C:N ratios. Pair-wise tests were carried out if significant differences occurred ($P < 0.05$). PERMANOVA tests were performed in the software PRIMER 6+ (PRIMER-E Ltd, Plymouth, UK).

References

1. de Fommervault OP, D'Ortenzio F, Mangin A, Serra R, Migon C, Claustre H, et al. Seasonal variability of nutrient concentrations in the Mediterranean Sea: Contribution of Bio-Argo floats. Journal of Geophysical Research: Oceans 2015;120:8528-8550.

2. Danovaro R. Detritus-bacteria-meiofauna interactions in a seagrass bed (*Posidonia oceanica*) of the NW mediterranean. Mar Biol 1996;127:1-13.

3. López NI, Duarte CM, Vallespinós F, Romero J, Alcoverro T. Bacterial activity in NW Mediterranean seagrass (*Posidonia oceanica*) sediments. J Exp Mar Biol Ecol 1995;187:39-49.

4. Abadie A, Borges AV, Champenois W, Gobert S. Natural patches in *Posidonia oceanica* meadows: the seasonal biogeochemical pore water characteristics of two edge types. Mar Biol 2017;164:166.

5. Klawonn I, Lavik G, Böning P, Marchant H, Dekaezemacker J, Mohr W, et al. Simple approach for the preparation of $^{15\text{-}15}N_2$-enriched water for nitrogen fixation assessments: evaluation, application and recommendations. Frontiers in Microbiology 2015;6:769.

6. Mohr W, Großkopf T, Wallace DWR, LaRoche J. Methodological underestimation of oceanic nitrogen fixation rates. PLoS ONE 2010;5:e12583.

7. Dabundo R, Lehmann MF, Treibergs L, Tobias CR, Altabet MA, Moisander PH, et al. The contamination of commercial $^{15}N_2$ gas stocks with ^{15}N–labeled nitrate and ammonium and consequences for nitrogen fixation measurements. PLoS ONE 2014;9:e110335.

8. van Kessel MAHJ, Speth DR, Albertsen M, Nielsen PH, Op den Camp HJM, et al. Complete nitrification by a single microorganism. Nature 2015;528:555.

9. Montoya JP, Voss M, Kahler P, Capone DG. A Simple, high-precision, high-sensitivity tracer assay for N_2 fixation. Appl Environ Microbiol 1996;62:986-993.

10. Di HJ, Cameron KC, McLaren RG. Isotopic dilution methods to determine the gross transformation rates of nitrogen, phosphorus, and sulfur in soil: a review of the theory, methodologies, and limitations. Soil Research 2000;38:213-230.

11. Petersen JM, Kemper A, Gruber-Vodicka H, Cardini U, van der Geest M, Kleiner M, et al. Chemosynthetic symbionts of marine invertebrate animals are capable of nitrogen fixation. Nat Microbiol 2016;2:16195.

12. Kirkham D, Bartholomew W. Equations for following nutrient transformations in soil, utilizing tracer data 1. Soil Sci Soc Am J 1954;18:33-34.

13. Jackson AL, Inger R, Parnell AC, Bearhop S. Comparing isotopic niche widths among and within communities: SIBER – Stable Isotope Bayesian Ellipses in R. J Anim Ecol 2011;80:595-602.

14. R Development Core Team. 2016 R: A language and environment for statistical computing. (R Foundation for Statistical Computing, Vienna, Austria.

15. Newsome SD, Martinez del Rio C, Bearhop S, Phillips DL. A niche for isotopic ecology. Frontiers in Ecology and the Environment 2007;5:429-436.

16. Yeakel JD, Bhat U, Elliott Smith EA, Newsome SD. Exploring the isotopic niche: isotopic variance, physiological incorporation, and the temporal dynamics of foraging. Frontiers in Ecology and Evolution 2016;4.

17. Bearhop S, Adams CE, Waldron S, Fuller RA, Macleod H. Determining trophic niche width: a novel approach using stable isotope analysis. J Anim Ecol 2004;73:1007-1012.

18. Radice VZ, Hoegh-Guldberg O, Fry B, Fox MD, Dove SG. Upwelling as the major source of nitrogen for shallow and deep reef-building corals across an oceanic atoll system. Funct Ecol 2019;doi:10.1111/1365-2435.13314.

19. Anderson MJ. A new method for non-parametric multivariate analysis of variance. Austral Ecol 2001;26:32-46.
